# Long-Term Follow-Up of Fractional CO_2_ Laser Therapy for Genitourinary Syndrome of Menopause in Breast Cancer Survivors

**DOI:** 10.3390/jcm11030774

**Published:** 2022-01-31

**Authors:** Allison M. Quick, Andrew Hundley, Cynthia Evans, Julie A. Stephens, Bhuvaneswari Ramaswamy, Raquel E. Reinbolt, Anne M. Noonan, Jeffrey Bryan Van Deusen, Robert Wesolowski, Daniel G. Stover, Nicole Olivia Williams, Sagar D. Sardesai, Stephanie S. Faubion, Charles L. Loprinzi, Maryam B. Lustberg

**Affiliations:** 1 Department of Radiation Oncology, The Ohio State University Medical Center, Columbus, OH 43210, USA; 2 Department of Obstetrics and Gynecology, The Ohio State University Medical Center, Columbus, OH 43210, USA; andrew.hundley@osumc.edu (A.H.); cynthia.evans@osumc.edu (C.E.); 3 The Ohio State University Center for Biostatistics, Columbus, OH 43210, USA; julie.stephens@osumc.edu; 4 Division of Medical Oncology, The Ohio State University Medical Center, Columbus, OH 43210, USA; bhuvaneswari.ramaswamy@osumc.edu (B.R.); raquel.reinbolt@osumc.edu (R.E.R.); anne.noonan@osumc.edu (A.M.N.); jeffrey.vandeusen@osumc.edu (J.B.V.D.); robert.wesolowski@osumc.edu (R.W.); daniel.stover@osumc.edu (D.G.S.); nicole.williams@osumc.edu (N.O.W.); sagar.saredesai@osumc.edu (S.D.S.); 5 Department of Medicine, Mayo Clinic, Jacksonville, FL 32256, USA; faubion.stephanie@mayo.edu; 6 Division of Medical Oncology, Mayo Clinic, Rochester, MN 55902, USA; cloprinzi@mayo.edu; 7 Division of Medical Oncology Yale Cancer Center, New Haven, CT 06520, USA; maryam.lustberg@yale.edu

**Keywords:** vaginal atrophy, genitourinary syndrome of menopause, breast cancer, fractional CO_2_ laser therapy

## Abstract

(1) Background: The objective of this study was to determine the long-term efficacy of fractional CO_2_ laser therapy in breast cancer survivors. (2) Methods: This was a single-arm study of breast cancer survivors. Participants received three treatments of fractional CO_2_ laser therapy and returned for a 4 week follow-up. Participants were contacted for follow-up at annual intervals. The Vaginal Assessment Scale (VAS), the Female Sexual Function Index (FSFI), the Female Sexual Distress Scare Revised (FSDS-R), the Urinary Distress Inventory (UDI), and adverse events were collected and reported for the two-year follow-up. The changes in scores were compared between the four-week and two-year and the one-year and two-year follow-ups using paired *t*-tests. (3) Results: In total, 67 BC survivors were enrolled, 59 completed treatments and the four week follow-up, 39 participated in the one-year follow-up, and 33 participated in the two-year follow-up. After initial improvement in the VAS from baseline to the four week follow-up, there was no statistically significant difference in the VAS score (mean Δ 0.23; 95% CI [−0.05, 0.51], *p* = 0.150) between the four week follow-up and the two-year follow-up. At the two-year follow-up, the FSFI and FSDS-R scores remained improved from baseline and there was no statistically significant change in the FSFI score (mean Δ −0.83; 95% CI [−3.07, 2.38] *p* = 0.794) or the FSDS-R score (mean Δ −2.85; 95% CI [−1.88, 7.59] *p* = 0.227) from the one to two-year follow-up. The UDI scores approached baseline at the two-year follow-up; however, the change between the one- and two-year follow-ups was not statistically significant (mean Δ 4.76; 95% CI [−1.89, 11.41], *p* = 0.15). (4) Conclusions: Breast cancer survivors treated with fractional CO_2_ laser therapy have sustained improvement in sexual function two years after treatment completion, suggesting potential long-term benefit.

## 1. Introduction

Genitourinary syndrome of menopause (GSM) is a constellation of symptoms that affect many breast cancer survivors due to the direct effect of cancer treatments, from change in menopausal status related to treatment, or from discontinuation of the use of hormone therapy because of their diagnosis [1,2,3]. Symptoms of GSM include vaginal dryness and dyspareunia that can affect long-term quality of life [1,4,5]. The vaginal changes from estrogen deprivation after menopause can lead to chronic symptoms that worsen over time without treatment [3,6]. While low-dose vaginal estrogens are a treatment approach for postmenopausal women, concerns about systemic absorption limit their use in the breast cancer population [6].

Fractional CO_2_ laser therapy is a new modality that has been used to treat GSM in postmenopausal women [7,8,9,10,11,12], with results similar to those observed with vaginal estrogen treatment [13,14,15,16]. Fractional CO_2_ laser therapy remodels vaginal tissue by activation of fibroblasts and increasing collagen production and neovascularization [17] through direct controlled thermal effect on the vaginal mucosa [18]. Two recent small, randomized sham-controlled studies showed improvement in GSM symptoms with laser treatment compared to sham treatment [19,20]. Fractional CO_2_ laser treatment has also been used in women with cancer, most commonly breast cancer. Data based on retrospective [21,22,23] and several small single-arm studies have shown improvement in vaginal symptoms and sexual function [24,25,26,27,28]. A major limitation of these studies has been a lack of long-term follow-up to evaluate persistence of benefit of this intervention.

We conducted a pilot study of fractional CO_2_ laser treatment in breast cancer survivors with GSM and demonstrated feasibility based on 88% of those enrolled completing all treatments according to protocol without serious adverse events. In addition, the treatment appeared to reduce symptoms of GSM at the initial one-month follow-up [25]. This study protocol was amended to collect follow-up data at one and two years to augment the limited long-term data on this treatment. Patient-reported sexual function was collected at one-year and demonstrated improvement in the FSFI and FSDS-R scores [26]. Here, we report the two-year follow-up results.

## 2. Materials and Methods

### 2.1. Sample and Eligibility

Women with non-metastatic breast cancer with symptoms of GSM including dyspareunia and/or vaginal dryness were recruited for this study as previously reported [25]. Patients had completed surgery, chemotherapy, and/or radiation for their breast cancer. They may have been on endocrine therapy and/or trastuzumab at the time of this study.

Exclusion criteria for this study were metastatic breast cancer, vaginal stenosis preventing placement of the vaginal probe, active urogenital infection, stage II or higher pelvic organ prolapse, a history of mesh-based reconstructive pelvic surgery, and systemic estrogen therapy or vaginal estrogen or dehydroepiandrosterone within six weeks prior to enrollment.

Women meeting the eligibility criteria and willing to participate consented for treatment. This study and the amendment were approved by The Ohio State University Cancer Institutional Review Board in July 2017 and May 2019, respectively. All procedures performed in studies involving human participants were in accordance with the ethical standards of the institutional research committee and with the 1964 Helsinki declaration and its later amendments or comparable ethical standards.

### 2.2. Procedures

Demographics, clinical data, and inclusion/exclusion criteria were obtained for all participants and have been previously reported [25]. All women were contacted for two-year follow-up either by phone or during a routine clinic visit to medical oncology or urogynecology. Three attempts were made by phone for follow-up assessment before classifying the participant as lost to follow-up. Subjective and objective symptoms of GSM were assessed at baseline and the 4 week follow-up. Subjective symptoms were also assessed at the two-year follow-up. Additional questionnaires assessing sexual function and urinary function were assessed at the same time points and were also collected at one-year follow-up. Adverse events (AE) were also solicited.

The treatment protocol which consisted of fractional microablative CO_2_ laser (MonaLisa Touch^TM^, DEKA Florence, Italy) at three time points 30–45 days apart has been previously described in detail [25]. The vaginal probe was inserted and laser pulses were delivered to the entire circumference and length of the vagina. The vestibule and posterior fourchette were treated using an external probe. For 48 h prior to and following the procedure, participants were informed to abstain from sexual intercourse and the use of topical vaginal products.

### 2.3. Measures

Subjective vaginal symptoms: The Vaginal and Vulvar Assessment Scales (VAS and VuAS), validated clinical measurement tools where a lower score indicated better function, were used to assess vaginal symptoms and dyspareunia over the preceding 4 weeks [29,30]. Because the VAS was designed as a physician-reported measure, it was initially not collected at the one-year follow-up since many participants were contacted via phone. With permission of the developer of the VAS [30], we used a patient-reported version of the questionnaire with the same questions and collected this information at the two-year follow-up.

Sexual function and distress: Sexual function was measured using the Female Sexual Function Index (FSFI), a 19 item self-reported instrument that measures six domains of sexual functioning and has been validated in breast cancer survivors [31]. A score of ≤26.55 indicates risk for sexual dysfunction, with higher scores indicating better sexual function [32]. The Female Sexual Distress Scale-Revised (FSDS-R) is a 13-item self-reported instrument designed to measure sexually-related psychological distress in women [33]. A higher score indicates more sexual distress and a total score of ≥11 shows high sensitivity (93%), specificity (96%), and positive predictive value (87.5%) for low libido after menopause [33,34].

Urinary function: Urinary symptoms were assessed using the Urogenital Distress Inventory-6 (UDI-6), a validated 6 item questionnaire with three subscales with scores that are added for a total score, with a higher score indicating higher disability [35]. The average score was calculated for the answered items only. The average UDI was multiplied by 25 to scale the total to a score of 100. If a subject answered “No” for the first question and “Not at All” for the second question, they were given a score of zero.

Adverse Events: AE were self-reported prior to each treatment and at the 4 week, one-year and two-year follow-ups. They were graded according to the NCI Common Terminology Criteria for Adverse Events v4.0 (CTCAE).

Statistical analysis: Estimates were calculated on the subset of participants who agreed to participate in the two-year follow-up. Descriptive statistics including means and 95% confidence intervals for continuous variables and proportions for categorical variables were used to summarize demographics, clinical characteristics and endpoints. The changes in endpoints from the four-week and one-year to the two-year follow-up were compared using paired *t*-tests. Although we present *p*-values in this paper, we consider these to be descriptive and hypothesis generating. All analyses were performed using SAS Version 9.4 (SAS Inc., Cary, NC, USA). All statistical tests were two sided with a 0.05 level of significance.

## 3. Results

**Figure 1:**
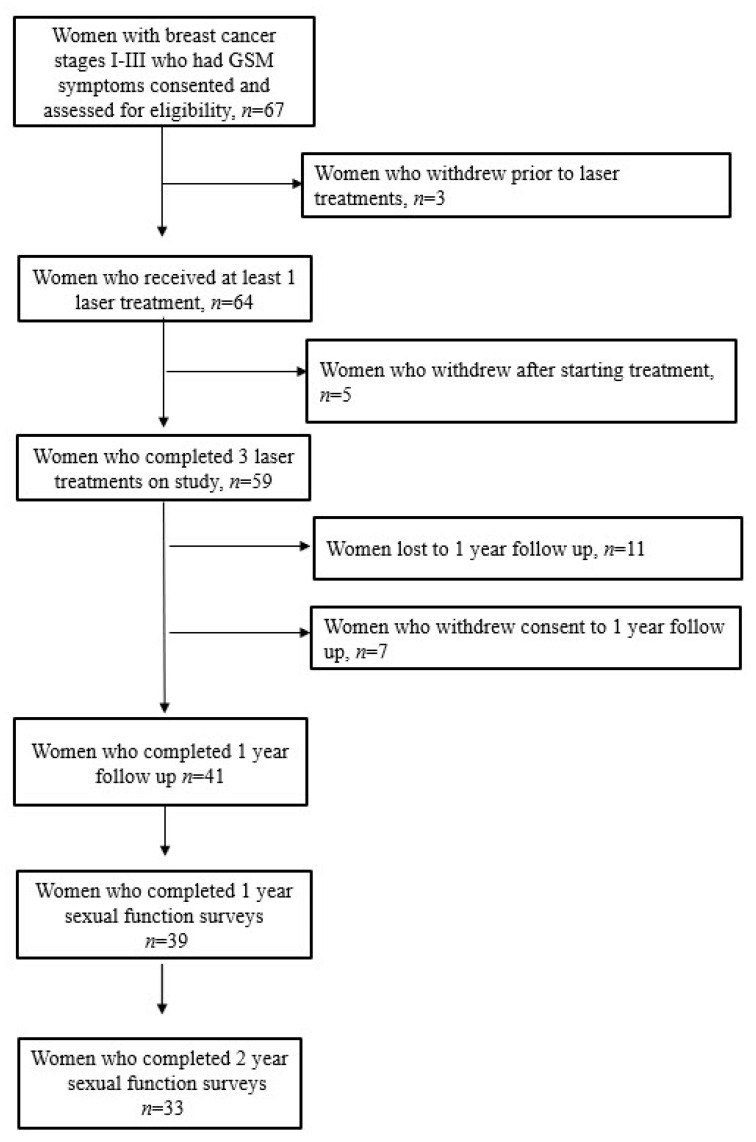
Consort diagram including long-term follow-up of study participants. GSM = Genitourinary syndrome of menopause.

A total of 67 patients enrolled in the initial pilot study and 3 withdrew from this study at screening, resulting in a total of 64 treated participants. Five patients withdrew after starting treatment. After extending follow-up of the study participants, 41 of the initial 59 women agreed to participate in the one-year follow-up and 33 were able to be contacted and agreed to participate in the two-year follow-up (Figure 1). Participant and breast cancer treatment characteristics are summarized in Table 1.

### 3.1. VAS/VuAS

We previously demonstrated that there was a statistically significant decrease in the total VAS score from baseline to the four-week follow-up, indicating an improvement in vaginal symptoms [25]. With long-term follow-up, there was a slight increase in the mean VAS score (*n* = 31) from the four-week follow-up to the two-year follow-up but it was not statistically significant (mean Δ 0.23; 95% CI [−0.05, 0.51], *p* = 0.150).

**Figure 2:**
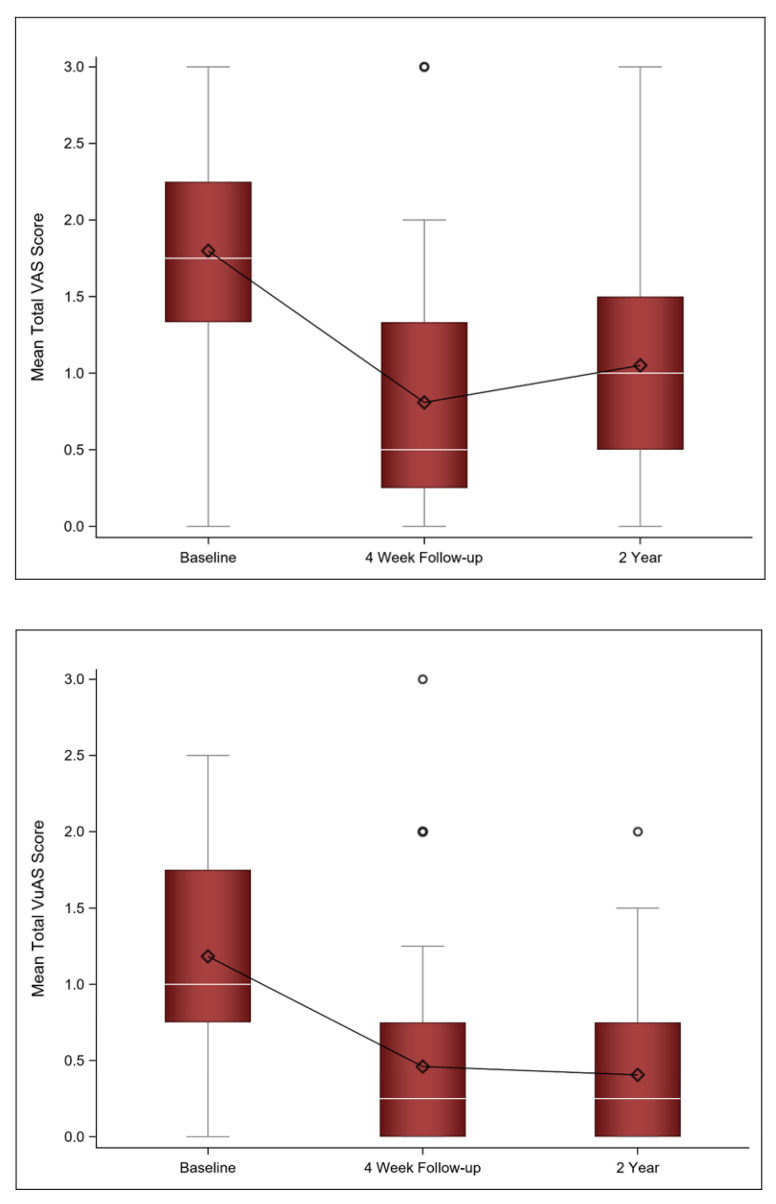
Mean (diamond), median (white line), and the 1st and 3rd quartiles are displayed for the VAS and VuAS at baseline, 4 week, and 2 year follow-up. The whiskers are the distance equal to 1.5 times the interquartile range (IQR). The diagram also shows outliers (circles) which are values that above or below the whisker ends. VAS = Vaginal Assessment Scale; VuAS = Vulvar Assessment Scale.

Similarly, there was initially a statistically significant improvement in the VuAS score from baseline to the four-week follow-up [25]. With additional long-term follow-up, there was no statistically significant difference between the four-week follow-up and the two-year follow-up scores (mean Δ −0.03; 95% CI [−0.33, 0.26] *p* = 0.817). Figure 2 shows VAS and VuAS scores at baseline, four-week follow-up, and two-year follow-up.

### 3.2. Sexual Function

**Figure 3:**
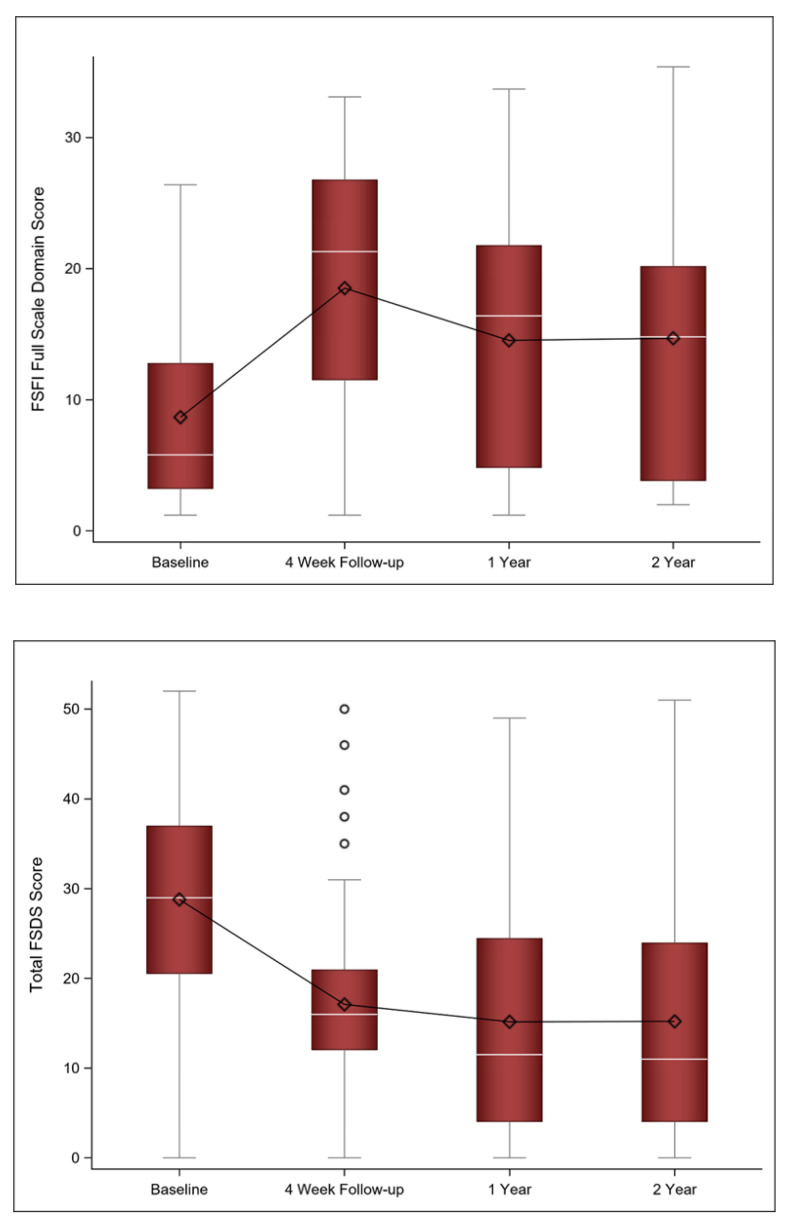
Mean (diamond), median (white line), and the 1st and 3rd quartiles are displayed for the FSFI and FSDS at baseline, 4 week, and 1 and 2 year follow-ups. The whiskers are the distance equal to 1.5 times the interquartile range (IQR). The diagram also shows outliers (circles) which are values that above or below the whisker ends. FSFI = Female Sexual Function Index; FSDS = Female Sexual Distress Scale.

At the two-year follow-up, the sexual function questionnaire scores remained improved from baseline and were stable from the one-year follow-up. There was no statistically significant change in the FSFI score (mean Δ −0.83; 95% CI [−3.07, 2.38] *p* = 0.794) or the FSDS-R scores (mean Δ −2.85; 95% CI [−1.88, 7.59] *p* = 0.227) from the one to the two-year follow-up. In fact, approximately 91% of participants had a FSDS-R score of ≥11 at baseline, indicating sexually-related distress, compared to 54% at the one-year follow-up and 52% at the two-year follow-up. Figure 3 shows the FSFI and FSDS scores at baseline as well as the four-week, one-year, and two-year follow-ups.

### 3.3. Urinary Symptoms

**Figure 4:**
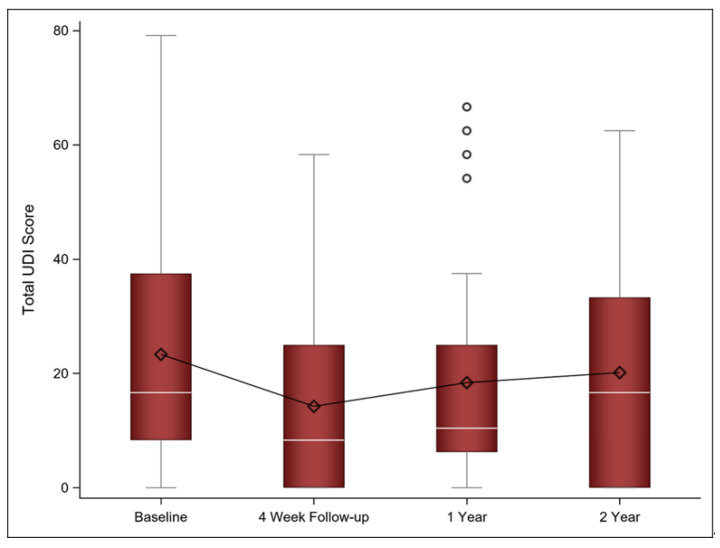
Mean (diamond), median (white line), and the 1st and 3rd quartiles are displayed for the UDI at baseline, 4 week, and 1 and 2 year follow-ups. The whiskers are the distance equal to 1.5 times the interquartile range (IQR). The diagram also shows outliers (circles) which are values that above or below the whisker ends. UDI = Urinary Distress Inventory.

While UDI scores decreased, suggesting improvement, at the initial four-week follow-up and one-year follow-up, they approached baseline at the two-year follow-up. However, the change between the one- and two-year follow-ups was not statistically significant (mean Δ 4.76; 95% CI [−1.89, 11.41], *p* = 0.155). Figure 4 shows the UDI scores at baseline as well as the four-week, one-year, and two-year follow-ups.

### 3.4. Adverse Events

No grade 3 or higher adverse events were identified at the two-year follow-up. 

## 4. Discussion

This study found that initial improvement in sexual function noted after fractional CO_2_ laser therapy in breast cancer survivors was sustained at two years post-treatment, in a subset of the initial study population. In contrast, the initial improvement in vaginal and urinary symptoms approached baseline levels after two years. This suggests the potential return of some symptoms over time without additional treatments. There was no SAE reported with long-term follow-up.

Approximately 50% of postmenopausal women will suffer symptoms of GSM that can be unrelenting without treatment [3]. Although vaginal dryness is typically the most bothersome symptom, it is often associated with sexual dysfunction [3]. The number of breast cancer survivors with GSM is higher than the general population of postmenopausal women, particularly breast cancer survivors on aromatase inhibitors (AI) [1,5,36,37]. Due to the severity of symptoms, some women discontinue AI therapy, potentially compromising their cancer outcomes [38]. Topical lubricants and moisturizers can be used for mild symptoms but symptoms often progress despite their use [3]. Low-dose vaginal estrogen use continues to be controversial in breast cancer survivors [39] but may have a role in some women with breast cancer with severe symptoms after close consultation with the oncologist for discussion of risk with treatment [40,41]. While permitted as second line therapy for dyspareunia by ESO-ESMO international consensus guidelines [42], topical hormone therapy did not improve vaginal symptoms or sexual problems in women with breast cancer in a recent retrospective study [43].

Laser treatments, including fractional CO_2_ laser therapy [11,12,15,18,44,45], erbium laser therapy (Er:YAG) [46,47], and other energy based devices, such as radiofrequency treatments [48], are being used to treatment symptoms of GSM. A meta-analysis of vaginal laser treatments [CO_2_ laser (*n* = 10 papers) and Er: YAG (*n* = 4 papers)] concluded that laser therapy appears to reduce symptoms of GSM and improve quality of life [44]. Similar to vaginal estrogen treatment, vaginal laser treatment has been shown to increase the thickness of vaginal epithelium and improve the quality of the vaginal mucosa [14]. However, there is still limited prospective data with long-term follow-up and there are some studies which failed to show an improvement with laser treatment [49,50].

The results of the current study are consistent with other prospective single-arm studies of fractional CO_2_ laser therapy in postmenopausal women and women with breast cancer. There are very few studies with long-term follow-up, however, limiting knowledge about duration of treatment effects and long-term side effects. A prospective single-arm study by Veron et al. demonstrated improvement in sexual and urinary function with laser treatment in breast cancer survivors (*n* = 46). The improvement in sexual function persisted at longer-term follow-up of 18 months. There was no patient-reported vaginal dryness or other vaginal symptoms reported [28]. The current study is the first to report follow-up at two years and supports sustained benefit in sexual function but suggests the possibility that the vaginal and urinary symptom benefit may be of shorter duration and may require additional treatments beyond one year.

Sham-controlled studies have mixed results. A small randomized double-blind, sham-controlled study in 30 postmenopausal women did not demonstrate a difference in vaginal symptoms, sexual function, or urinary symptoms between the treatment arm (*n* = 14) and the sham-controlled arm (*n* = 16). However, the study was likely underpowered due to its small sample size [49]. Another recently published sham-controlled randomized trial of 85 postmenopausal women, 50% of whom had a history of breast cancer, also showed no improvement in vaginal symptoms with laser treatment compared to sham treatment with 12 months follow-up [50]. However, a randomized double-blind, sham-controlled study of 58 postmenopausal women by Salvatore et al. showed that vaginal symptoms and FSFI and UDI-6 scores were improved in the treatment group compared to the sham group [19]. Another randomized sham-controlled pilot study was also conducted in women with gynecologic cancers which showed that laser treatments were safe and appeared to improve sexual function based on FSFI scores [51].

While the majority of studies have used three laser treatments, the exact number of treatments needed to achieve and maintain benefit remains unknown. A recent small prospective pilot study of 40 women with breast cancer delivered five laser treatments every four weeks and were re-evaluated at a four-week follow-up. The majority of women were satisfied with treatment and experienced improvement in subjective and objective symptoms of vulvovaginal atrophy, quality of life, and sexual function [52].

The strength of the current study is that it is has the longest follow-up of any the reported study on fractional CO_2_ laser therapy, including postmenopausal women or cancer survivors, which continues to support the lack of long-term adverse event from laser treatment.

The limitations of the study include lack of a sham comparison arm and the loss of a significant number of the treated women resulting in bias in the scores of the subjects who agreed to participate through two-year follow-up, compared to those who did not. Additionally, this study was not powered to detect differences at the two-year follow-up. Therefore, a double-blind, randomized controlled trial, comparing fractional CO_2_ laser therapy versus sham laser treatment is needed to further establish the short- and long-term safety and efficacy of CO_2_ laser treatment in women with breast cancer.

## 5. Conclusions

Breast cancer survivors treated with fractional CO_2_ laser therapy reported sustained improvement in sexual function with no serious adverse effects two years after initial treatment in this limited study.

## Figures and Tables

**Table 1:** Participant and breast cancer treatment characteristics.

	Initial Participants with 4 Week Follow-Up (*n* = 64)	Participants with 1 Year Follow-Up (*n* = 39)	Participants with 2 Year Follow-Up (*n* = 33)
Age (on study)			
Mean (SD)	57.4 (9.5)	57.7 (10.50)	59.3 (10.8)
Stage			
I	32 (50.0%)	19 (48.7%)	15 (45.5%)
II	21 (32.8%)	13 (33.3%)	10 (30.3%)
III	7 (10.9%)	4 (10.3%)	5 (15.2%)
IV	1 (1.6%) *b*	1 (2.6%)	0 (0%)
Histology			
Adenocarcinoma	51 (76.7%)	32 (82.1%)	27 (81.8%)
Other	13 (20.3%)	7 (18.0%)	6 (18.2%)
ER status			
Negative	5 (7.8%)	0 (0%)	1 (3.0%)
Positive	58 (90.6%)	38 (97.4%)	31 (93.9%)
Unknown	1 (1.6%)	1 (2.6%)	1 (3.0%)
PR status			
Negative	15 (23.4%)	6 (15.4%)	7 (21.2%)
Positive	47 (73.4%)	31 (79.5%)	25 (75.8%)
Unknown	2 (3.1%)	2 (5.1%)	1 (3.0%)
HER2 Neu status			
Negative	50 (78.1%)	28 (71.8%)	26 (78.8%)
Positive	11 (17.2%)	9 (23.1%)	4 (12.1%)
Unknown	3 (4.7%)	2 (5.1%)	3 (9.1%)
ER−/PR−	5 (7.8%)	0 (0%)	1 (3.0%)
ER+/PR+	47 (73.4%)	31 (79.5%)	25 (75.8%)
ER+/PR−	10 (15.6%)	6 (15.4%)	6 (18.2%)
Chemotherapy			
Yes	36 (56.3%)	20 (51.3%)	16 (48.5%)
No	27 (42.2%)	18 (46.2%)	16 (48.5%)
Unknown	1 (1.6%)	1 (2.6%)	1 (3.0%)
Endocrine therapy use			
Yes	59 (92.2%)	37 (94.9%)	32 (97.0%)
No	4 (6.3%)	1 (2.6%)	0 (0%)
Unknown	1 (1.6%)	1 (2.6%)	1 (3.0%)
Type of endocrine therapy			
Tamoxifen	13 (20.3%)	9 (23.1%)	7 (21.2%)
AI	44 (68.8%)	26 (66.7%)	23 (69.7%)
Tamoxifen and ovarian suppression	2 (3.1%)	2 (5.1%)	2 (6.1%)

SD = standard deviation; ER = estrogen receptor; PR = progesterone receptor, HER2 = human epidural growth factor receptor 2; AI = aromatase inhibitor.

## Data Availability

The corresponding author has full control of the primary data and can be reviewed by the journal if requested.
